# Complete Genome Sequences of Extremely Thermoacidophilic Metal-Mobilizing Type Strain Members of the Archaeal Family Sulfolobaceae, Acidianus brierleyi DSM-1651, Acidianus sulfidivorans DSM-18786, and Metallosphaera hakonensis DSM-7519

**DOI:** 10.1128/MRA.00831-18

**Published:** 2018-07-19

**Authors:** James A. Counts, Nicholas P. Vitko, Robert M. Kelly

**Affiliations:** a Department of Chemical and Biomolecular Engineering, North Carolina State University, Raleigh, North Carolina, USA; University of Arizona

## Abstract

The family Sulfolobaceae contains extremely thermoacidophilic archaea that are found in terrestrial environments. Here, we report three closed genomes from two currently defined genera within the family, namely, Acidianus brierleyi DSM-1651^T^, Acidianus sulfidivorans DSM-18786^T^, and Metallosphaera hakonensis DSM-7519^T^.

## ANNOUNCEMENT

Members of the crenarchaeal family Sulfolobaceae are exclusively extreme thermoacidophiles, given that their optimal growth temperatures exceed 65˚C and their optimal pH levels are below 3.5. Thus, they inhabit the most inhospitable environments on earth (e.g., volcanic solfatara fields, geothermal mud springs, etc.) (1). Three species, Acidianus brierleyi (2), Acidianus sulfidivorans (3), and Metallosphaera hakonensis (4), were identified as candidates for sequencing given their reported abilities to utilize metal substrates as electron donors and support chemolithoautotrophic growth. The family Sulfolobaceae, within the order Sulfolobales, was originally named for its members’ perceived ability to utilize, and thrive in, sulfur-rich thermal environments; newer evidence, however, shows that, in fact, many members of this taxonomical clade fail to utilize sulfur and/or other sources of electron donors, including metals and their ores, as well as simple and complex carbohydrates. The source locations and known optimal growth conditions for these species are summarized in Table 1.

**TABLE 1:** Characteristics of sequenced species and genome sequence assembly statistics^*a*^

Species	DSM/JCM designation	Isolation site	*T*_opt_ (°C)	pH_opt_	G+C content (mol%)	Genome size (bp)	No. of CDSs	No. of tRNAs	No. of rRNAs	GenBank accession no.
A. brierleyi	1651/8954	Acidic hot spring, Yellowstone NP, USA	70	1.5–2.0	31 (2)	2,947,156	3,120	46	3	CP029289
A. sulfidivorans	18786/13667	Solfatara, Lihir Island, Papua New Guinea	74	0.8–1.4	31.1 (3)	2,287,077	2,312	46	3	CP029288
M. hakonensis	7519/8857	Geothermal field, Hakone NP, Japan	70	3.0	46.2 (7)	2,544,018	2,570	45	3	CP029287

a Abbreviations: *T*_opt_, optimal temperature for growth; pH_opt_, optimal pH level for growth; CDSs, coding sequences; NP, national park.

To date, there is limited genomic information available for extreme thermoacidophiles, partly due to difficulties in assembling their genome sequences. As an example, a previous sequencing of M. hakonensis via the Ion PGM platform resulted in an assembly of 129 contigs (total size, 2,387,907 bp; largest contig size, 269,819 bp; *N*_50_ contig size, 59,396 bp; RefSeq assembly accession number GCF_001315825.1). Here, single-molecule real-time (SMRT) technology was used to overcome previous assembly limitations.

Each of the species, whose genome sequences are reported here, was obtained from the Leibniz-Institut DSMZ GmbH. Cultures were grown at their optimal conditions (as specified by DSMZ), and genomic DNA was isolated using phenol-chloroform-isoamyl alcohol separation and propanol precipitation. Samples were sequenced using a PacBio RS II system with one SMRT cell per organism. The assembly was created using CLC Genomics version 11.0.1 with the proprietary Genome Finishing Module, which uses a de Bruijn graph algorithm tailored to PacBio data to improve the final output (5). Long read data (with coverage in excess of 300×) were corrected with the software (100× coverage retained) and used for the assembly, after which the contigs were joined via an iterative process of manual curation (mapping, correcting, extending, and aligning). All parameters, except the percentage of reads to retain during the correction step, were default values. A summary of the final statistics is given in Table 1. Once closed, the assemblies were annotated using the NCBI Prokaryotic Genome Annotation Pipeline (PGAP) (6).

Overall, the assembled genome sequences represent both new additions to the available genome sequence information on species representing two of the genera within the family Sulfolobaceae and an improvement on the previously reported assembly of M. hakonensis.

### Data availability.

The genome sequence information reported here has been deposited in DDBJ/ENA/GenBank under the accession numbers given in Table 1 and under the BioProject ID PRJNA463410.

## References

[1] Wheaton G, Counts J, Mukherjee A, Kruh J, Kelly R 2015 The confluence of heavy metal biooxidation and heavy metal resistance: implications for bioleaching by extreme thermoacidophiles. Minerals 5:397–451. doi:10.3390/min5030397.

[2] Segerer A, Neuner A, Kristjansson JK, Stetter KO 1986 *Acidianus infernus* gen. nov., sp. nov., and *Acidianus brierleyi* comb. nov.: facultatively aerobic, extremely acidophilic thermophilic sulfur-metabolizing archaebacteria. Int J Syst Bacteriol 36:559–564. doi:10.1099/00207713-36-4-559.

[3] Plumb JJ, Haddad CM, Gibson JA, Franzmann PD 2007 *Acidianus sulfidivorans* sp. nov., an extremely acidophilic, thermophilic archaeon isolated from a solfatara on Lihir Island, Papua New Guinea, and emendation of the genus description. Int J Syst Evol Microbiol 57:1418–1423. doi:10.1099/ijs.0.64846-0. 17625168

[4] Takayanagi S, Kawasaki H, Sugimori K, Yamada T, Sugai A, Ito T, Yamasato K, Shioda M 1996 *Sulfolobus hakonensis* sp. nov., a novel species of acidothermophilic archaeon. Int J Syst Bacteriol 46:377–382. doi:10.1099/00207713-46-2-377. 8934897

[5] Pevzner PA, Tang H, Waterman MS 2001 An Eulerian path approach to DNA fragment assembly. Proc Natl Acad Sci U S A 98:9748–9753. doi:10.1073/pnas.171285098. 11504945 PMC55524

[6] Angiuoli SV, Gussman A, Klimke W, Cochrane G, Field D, Garrity G, Kodira CD, Kyrpides N, Madupu R, Markowitz V, Tatusova T, Thomson N, White O 2008 Toward an online repository of standard operating procedures (SOPs) for (meta)genomic annotation. OMICS 12:137–141. doi:10.1089/omi.2008.0017. 18416670 PMC3196215

[7] Kurosawa N, Itoh YH, Itoh T 2003 Reclassification of *Sulfolobus hakonensis* Takayanagi *et al.* 1996 as *Metallosphaera hakonensis* comb. nov. based on phylogenetic evidence and DNA G+C content. Int J Syst Evol Microbiol 53:1607–1608. doi:10.1099/ijs.0.02716-0. 13130056

